# Potential efficacy of digital polymerase chain reaction for non-invasive prenatal screening of autosomal aneuploidies: a systematic review and meta-analysis

**DOI:** 10.1186/s12884-024-06655-0

**Published:** 2024-07-11

**Authors:** Mohammadamin Parsaei, Mohadese Dashtkoohi, Tayyeb Ali Salmani, Mohammad Sadeq Najafi, Mohammad Haddadi, Marjan Ghaemi, Sedigheh Hantoushzadeh

**Affiliations:** 1 Maternal, Fetal & Neonatal Research Center, Family Health Research Institute, Imam Khomeini Hospital Complex, Tehran University of Medical Sciences, Tehran, Iran; 2 Breastfeeding Research Center, Family Health Research Institute, Imam Khomeini Hospital Complex, Tehran University of Medical Sciences, Tehran, Iran; 3 Vali-E-Asr Reproductive Health Research Center, Family Health Research Institute, Imam Khomeini Hospital Complex, Tehran University of Medical Sciences, District 6, Keshavarz Blvd, Gharib St, Tehran, Iran; 4 FETUS Medical Genetic Laboratory, Tehran, Iran; 5 Research Center for Advanced Technologies in Cardiovascular Medicine, Cardiovascular Diseases Research Institute, Tehran University of Medical Sciences, Tehran, Iran

**Keywords:** Down syndrome, Edwards syndrome, Noninvasive prenatal testing, Patau syndrome, Polymerase chain reaction

## Abstract

**Background:**

Digital Polymerase Chain Reaction (dPCR) presents a promising approach for quantifying DNA and analyzing copy number variants, particularly in non-invasive prenatal testing. This method offers a streamlined and time-efficient procedure in contrast to the widely used next-generation sequencing for non-invasive prenatal testing. Studies have reported encouraging results for dPCR in detecting fetal autosomal aneuploidies. Consequently, this systematic review aimed to evaluate the effectiveness of dPCR in screening for trisomy 21, 18, and 13.

**Methods:**

A systematic search was conducted in PubMed, Web of Sciences, and Embase for relevant articles published up to December 30, 2023. The Quality Assessment of Diagnostic Accuracy Studies-2 (QUADAS-2) was utilized for the quality assessment of the included articles. Furthermore, a bivariate random-effect regression model was used to conduct a meta-analysis on the utility of dPCR for trisomy 21 screening.

**Results:**

A total of 9 articles were included in this review, with all of them assessing the utility of dPCR in trisomy 21 screening, and 2 and 1 studies conducting additional analysis on the screening abilities of dPCR for trisomy 18 and 13, respectively. A bivariate random-effects model calculated pooled sensitivity and specificity with a 95% confidence interval (CI). Meta-analysis of 6 studies comparing trisomy-21 screening with karyotyping demonstrated dPCR's pooled sensitivity of 98% [95% CI: 94 -100] and specificity of 99% [95% CI: 99 -100]. While conducting a meta-analysis for trisomy 13 and 18 proved impractical, reported values for sensitivity and specificity were favorable.

**Conclusions:**

These findings suggest that dPCR holds promise as an effective tool for non-invasive prenatal testing, presenting a less time-consuming and intricate alternative to next-generation sequencing. However, further research is necessary to evaluate dPCR's applicability in clinical settings and to delineate its specific advantages over next-generation sequencing. This study contributes valuable insights into the potential of dPCR for enhancing prenatal screening methodologies.

**Trial registration:**

The protocol of this study was registered in the International Prospective Register of Systematic Reviews (PROSPERO) on 7/3/2024, with a registration code of CRD42024517523.

**Supplementary Information:**

The online version contains supplementary material available at 10.1186/s12884-024-06655-0.

## Background

Non-Invasive Prenatal Testing (NIPT), which relies on the analysis of cell-free fetal deoxyribonucleic acid (cff-DNA) in the maternal plasma to detect chromosomal abnormalities, has revolutionized prenatal screening for aneuploidies [1]. The cff-DNA screening by Next-Generation Sequencing (NGS) offers high sensitivity, specificity, positive predictive value, and a lower false positive rate compared to the traditional screening that involves measuring nuchal translucency and biochemical analytes [2, 3]. Previous studies have highlighted the efficacy of NGS in non-invasive prenatal screening for prevalent autosomal aneuploidies, namely trisomy 21 (Down’s syndrome), trisomy 18 (Edwards syndrome), and trisomy 13 (Patau syndrome) [4–6]. Although the NGS procedure is considered a breakthrough, it does have several disadvantages, including being time-consuming and costly [1, 7].

Digital polymerase chain reaction (dPCR) represents an innovative and fast method that enables more accurate quantification of target deoxyribonucleic acid (DNA) molecules through the partitioning of the polymerase chain reaction (PCR) into numerous discrete reactions [8]. Compared to traditional PCR methods, such as quantitative PCR (qPCR), dPCR offers absolute DNA quantification without necessitating standard curves [9, 10]. Notably, dPCR surpasses qPCR in analyzing copy number variants and is compatible with quantifying low-target levels in samples [9, 10]. This technology proves especially valuable when diluted samples would otherwise result in undetectable target levels using qPCR [9]. Furthermore, the reduced volume of required reagents contributes to the cost-effectiveness of this highly accurate method [11–13].

Recent high-quality studies examining the efficacy of dPCR in prenatal aneuploidy screening have reported favorable outcomes [14, 15]. Consequently, in the current investigation, we conducted a systematic review of published records to evaluate the applicability of dPCR in non-invasive prenatal screening for trisomy 21, 18, and 13. Additionally, a meta-analysis was undertaken to assess the utility of dPCR in trisomy 21 screening. Our review question was composed following the Patients, Intervention, Comparison, Outcome (PICO) guideline as follows: (1) Patients: pregnant women with fetal trisomy 21, 18, or 13, (2) Intervention: dPCR analysis on maternal serum cffDNA, (3) Comparison: results of the karyotyping analysis from invasive testing, and (4) Outcome: true positive (TP) rate, true negative (TN) rate, false positive (FP) rate, and false negative (FN) rate.

## Methods

### Search strategy

A systematic search was conducted in three electronic databases: PubMed, Embase, and Web of Science on December 30, 2023, for relevant articles on the utility of dPCR in prenatal diagnosis of either trisomy 21, 18, or 13. The search term consisted of two groups of keywords related to “dPCR” and “Aneuploidy”. Also, an additional manual search was conducted on the reference lists of the included studies and Google Scholar. Detailed information regarding the employed keywords and applied filters in each database are provided in Supplementary Material 1. This study was conducted following the Preferred Reporting Items for Systematic Reviews and Meta-Analysis (PRISMA) 2020 guidelines (Supplementary Material 2) [16]. The protocol of this study was registered in the International Prospective Register of Systematic Reviews (PROSPERO), with a registration code of CRD42024517523.

### Selection criteria

All peer-reviewed observational studies that evaluated the utility of dPCR in detecting either trisomy 21, 18, or 13 for non-invasive prenatal screening were considered eligible to be included. The exclusion criteria were: (a) records that did not employ digital PCR in their study, (b) records that did not assess either trisomy 21, 18, or 13, (c) records that were conducted on data other than prenatal samples, (d) records that included less than 10 prenatal samples, (e) non-human studies, and (f) review articles, case reports, conference abstracts, book chapters, letters, editorials, commentaries, correspondence, and study protocols.

### Data extraction

The following data were (if available) collected from each included article: (a) general study characteristics: first author, year of publication, country of origin, design (prospective or retrospective), (b) study sample characteristics: sample size, type of sample (maternal plasma, or whole-blood), maternal age, gestational age, pregnancy types, and estimated aneuploidy risk of the maternal population, (c) dPCR characteristics: device, primers (single-plex or multi-plex), and reaction protocol, (d) reference test, (e) determined diagnosis of samples (euploidy, trisomy 13, 18, or 21), and (f) diagnostic results of dPCR, including the reported values of the TN, TP, FN, and FP.

### Quality assessment

The Quality Assessment of Diagnostic Accuracy Studies-2 (QUADAS-2) [17] tool was utilized for the quality assessment. The QUADAS-2 statement systematically evaluates the risk of bias within four key domains: patient selection, index test, reference standard, and flow/timing. Additionally, the initial three domains are examined for the applicability of the study findings. Ratings of "low," "high," or "unclear" are ascribed to signify the perceived risk of bias and applicability within each respective QUADAS-2 domain. After this domain-specific evaluation, an overarching assessment of the overall risk of bias and applicability for each study is conducted by QUADAS-2 guidelines. The cumulative risk of bias for each study is categorized as either "low risk of bias" or "at risk of bias", while the overall applicability is categorized as either "low concerns regarding applicability" or "concerns regarding applicability".

### Meta-analysis

We also performed a meta-analysis to determine the diagnostic accuracy of dPCR for NIPT. For this aim, we pooled the data from studies that compared the screening results of dPCR with the results of karyotyping, which is known to be the gold standard for the diagnosis of fetal aneuploidies [18].

Statistical analyses were conducted by META-DISC 1.4 (Cochrane Colloquium) [19]. A bivariate random-effect regression model was used to estimate the pooled sensitivity, specificity, Positive Likelihood Ratio (PLR), Negative Likelihood Ratio (NLR), and Diagnostic Odds Ratio (DOR) with 95% Confidence Interval (CI). Also, the summary Receiver Operating Characteristics (sROC) was illustrated and the Area Under the Curve (AUC) was calculated for each study. Furthermore, the Inconsistency Index (*I*^2^), chi-square value, and p-values were calculated for each forest plot to determine the degree of heterogeneity across the studies. In this regard, an *I*^2^ > 50% was interpreted as high heterogeneity in the diagnostic parameter across the reviewed studies. Moreover, a *p*-value < 0.05 was considered to be significant.

Initially, we aimed to assess publication bias using Deek’s funnel plot asymmetry test in statistical software R version 4.0.3 (metafor package). A slope coefficient accompanied by a *p*-value > 0.10 was considered to indicate a high likelihood of publication bias across the included studies. However, given that the number of studies included in the meta-analyses was less than 10, we did not manage to illustrate Deek’s funnel plot [20]. Therefore, we conducted a leave-one-out sensitivity analysis to determine the consistency of the findings of the analyzed studies.

## Results

### Study characteristics

**Fig. 1:**
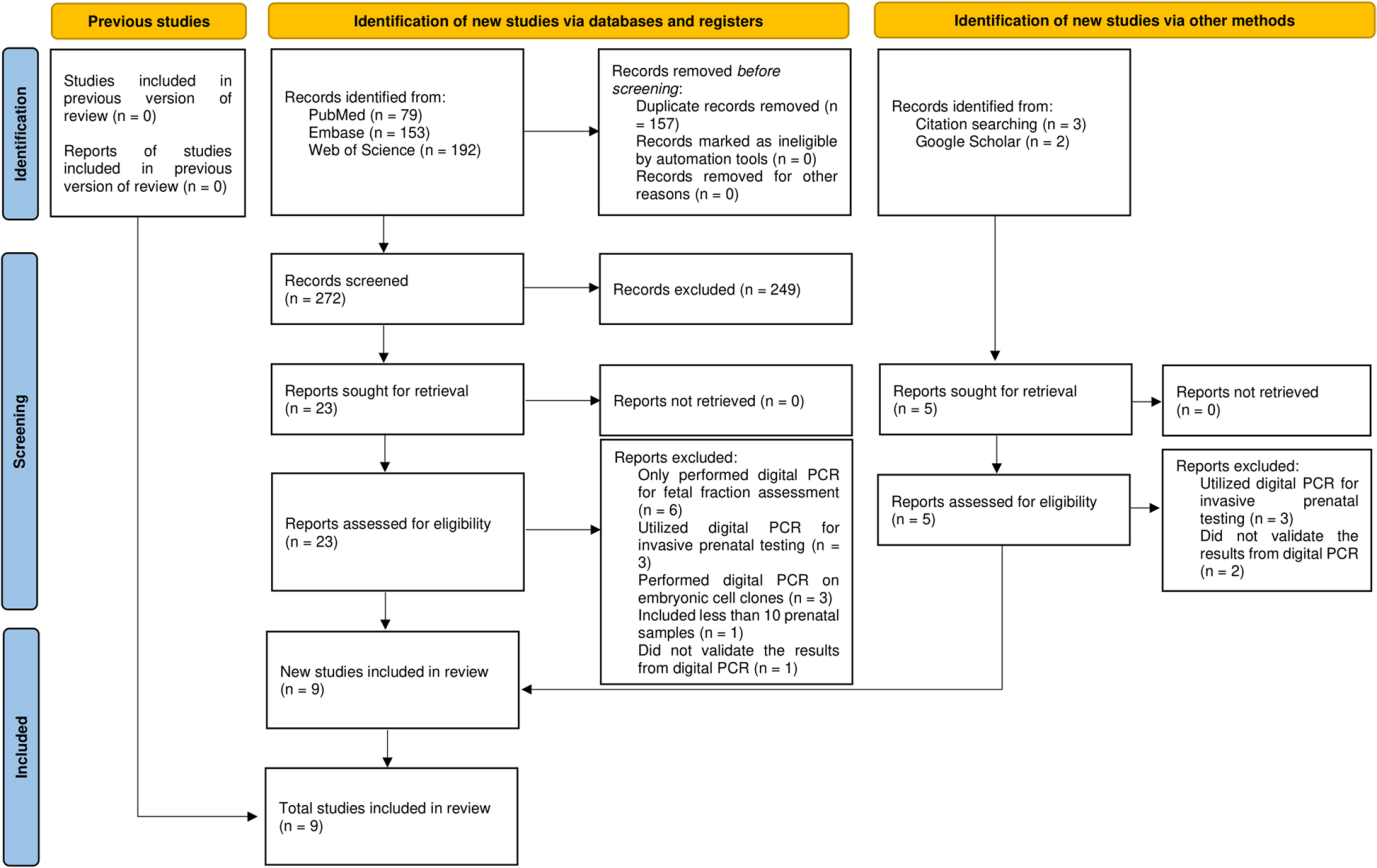
The flow chart of the study screening process, based on the PRISMA 2020 statement

Our search yielded a total of 424 records, with 5 additional records identified from the manual search. After removing the duplicates, 272 records underwent title and abstract screening which resulted in 23 articles for the full-text screening. Furthermore, 9 articles met the eligibility criteria and were included in this review. Figure 1 represents the flow chart for the study screening process.

The majority of the reviewed studies were from China (*n* = 5), with additional contributions from South Korea (*n* = 2), the Czech Republic (*n* = 1), and France (*n* = 1). Except for 2 studies with a retrospective design [21, 22], the other 7 studies were prospective. Collectively, a total of 1611 prenatal samples from either maternal plasma or whole blood were studied. Five studies compared the results of dPCR with findings of the karyotyping analysis on amniocentesis or Chorionic Villous Sampling (CVS) samples [22–26], and 1 compared it with karyotyping analysis or data from clinical follow-up [27]. Whereas 3 studies only compared dPCR results with NGS findings [21, 28, 29]. All of the included studies examined the utility of dPCR in trisomy 21 screening. Additionally, one study evaluated its applicability in trisomy18 screening [26], as well as one study assessing its accuracy in screening trisomy 13 and 18 [25]. Table 1 summarizes the characteristics and findings of each included article.

**Table 1:** Characteristics of the included studies^a^

Study	Country	Design	Population	Aneuploidy	Sample	Device	Chromosomal assay method	Target gene(s)	PCR conditions (reaction protocol)	Reference test
Lee (2015) [1]	South Korea	RC	Samples: (*n* = 43) Maternal age: NA; Gestational age: (11–24) w	Mixture	Maternal whole blood	QuantStudio™ 3D Digital PCR System	Multiplex	NA	95ºC hold step for 20 s, 40 cycles of PCR step including 1 s of 95ºC denature and 20 s of 60ºC annealing	NGS
El Khattabi (2016) [2]	France	PC	Samples: (*n* = 213) Maternal age: NA; Gestational age: 16 (9–37) w	High-risk	Maternal plasma	Bio-Rad QX100™ Droplet Digital PCR System	Multiplex	BRWD1, LTN1, NCAM2, and RUNX2	95ºC for 10 min, 40 cycles of 94ºC for 30 s and 60ºC for 1 min, and then final extension at 98ºC for 10 min	Karyotyping analysis
Xu (2016) [3]	China	RC	Samples: (*n* = 15) Maternal age: NA; Gestational age: NA	High-risk	Maternal plasma	QuantStudio™ 3D Digital PCR System	Single-plex	Segmental duplication	50ºC for 2 min and 95ºC for 2 min, followed by 50 cycles of 15 s at 95ºC, and 60 s at 60ºC	Karyotyping analysis
Lee (2018) [4]	South Korea	PC	Samples: (n = 877) Maternal age: (21–46) y; Gestational age: (10–20) w	Mixture	Maternal plasma	Bio-Rad QX200™ Droplet Digital PCR System	Multiplex	SETD4, CBR1, UBE2G2, and CLDN14	95ºC for 10 min, 40 cycles of 94ºC for 30 s and 60ºC for 1 min, and then final extension at 98ºC for 10 min	Karyotyping analysis, clinical follow-up
Wu (2018) [5]	China	PC	Samples: (*n* = 105) Maternal age: NA; Gestational age: (13–26) w	Mixture	Maternal plasma	Bio-Rad QX200™ Droplet Digital PCR System	Single-plex	HLCS	50ºC for 2 min, 95ºC for 10 min, 40 cycles of 94ºC for 15 s and 60ºC for 1 min	Karyotyping analysis
Tan (2019) [6]	China	PC	Samples: (*n* = 30) Maternal age: NA; Gestational age: 17.5 (12–25.3) w	Mixture	Maternal plasma	RainDrop™ Digital PCR System	Multiplex	NA	25 °C for 10 min, 95 °C for 10 min, 40 cycles of 94 °C for 20 s and 60 °C for 60 s, 72 °C for 5 min, 98 °C for 10 min, and finally held at 12 °C	NGS
Chen (2021) [7]	China	PC	Samples: (*n* = 15) Maternal age: NA; Gestational age: (14.1–19.7) w	Mixture	Maternal plasma	TargetingOne™ ddPCR System	Multiplex	Segmental duplication	Template denaturation at 95°C for 10 min, followed by 45 cycles of denaturation at 94°C for 30 s and annealing at 58°C for 60 s, and ended with heat preservation at 12°C	NGS
Dai (2022) [8]	China	PC	Samples: (*n* = 282) Maternal age: NA; Gestational age: 17 (12–36) w	High-risk	Maternal plasma	BioDigital- QING dPCR™ System	Multiplex	NA	95°C for 10 min, 6 cycles of 95°C for 30 s and 60°C for 30 s	Karyotyping analysis
Laššáková (2023) [9]	Czech Republic	PC	Samples (*n* = 30) Maternal age: NA; Gestational age: (13–18) w	High-risk	Maternal plasma	Nacia System™ Crystal Digital PCR Device	Multiplex	NA	95 °C for 5 min, followed by a two-stage touchdown PCR consisting of 35 cycles of 95 °C for 30 s, 63 °C for 90 s, then 15 cycles of 95 °C for 30 s, and 56 °C for 90 s	Karyotyping analysis

*Abbreviations*: *AC* Amniocentesis, *Chr* Chromosome, *CMA* Chromosome microarray analysis, *CVS* Chorionic villus sample, *dPCR* Digital PCR, *ddPCR* Digital droplet polymerase chain reaction, *n* Number, *NA* Not available, *NGS* Next-generation sequencing, *PC* Prospective cohort, *PCR* Polymerase chain reaction, *RC* Retrospective cohort, *w* Weeks, *y* Years

^a^Categorial data are presented as numbers and continuous data as mean or median ± standard deviation (range)

### Review of study findings

#### Trisomy 21

Lee et al. (2015) assessed dPCR against NGS for trisomy 21 detection in whole-blood samples from 33 low-risk mothers and 10 with confirmed fetal trisomy 21 diagnoses via karyotyping. Compared to NGS, dPCR successfully identified 9 out of 10 trisomy 21 cases with no reported FPs. However, the lone FN result had a notably lower cell-free fetal fraction ratio (0.79%) compared to other samples, which exceeded 2.5% [21].

El Khattabi et al. (2016) performed multiplex dPCR on plasma samples from 213 mothers labeled as high risk for fetal aneuploidies by targeting BRWD1, LTN1, NCAM2, and RUNX2 sites on chromosome 21. They compared the results with karyotyping analysis, and in this study, dPCR successfully identified all trisomy 21 cases without any FPs among the euploid cases [23].

Xu et al. (2016) employed dPCR to quantify segmental duplication in chromosome 21 for the NIPT of trisomy 21 in a population of mothers at high risk. They analyzed plasma samples from 15 mothers, 12 with a normal fetus and 3 with a trisomy 21 fetus as diagnosed by karyotyping analysis. In this study, dPCR successfully identified all trisomy 21 cases with no FP results [22].

Lee et al. (2018) devised a dPCR protocol for trisomy 21 detection, targeting SETD4, CBR1, UBE2G2, and CLDN14 on chromosome 21 using 160 samples from maternal plasma, whole blood, and amniotic fluid. They assessed the protocol on 877 clinical samples from a mixed-risk population of mothers, confirming dPCR results through karyotyping analysis or clinical follow-up. The findings revealed dPCR's success in detecting all 50 trisomy 21 cases, with only 3 reported FP results [27].

Wu et al. (2018) utilized dPCR to analyze maternal plasma samples, aiming to distinguish between 78 euploid fetal cases and 28 cases from mothers with confirmed fetal trisomy 21 diagnoses. By quantifying the ratio of the HLCS gene on chromosome 21 and the fetal-specific rs6636 SNP allele on chromosome 14, they successfully identified 25 out of 28 trisomy 21 cases with no reported FP results [24].

Tan et al. (2019) examined 30 maternal plasma samples with varying risks for fetal aneuploidies using multiplex dPCR and compared the results with NGS findings. Their findings revealed that dPCR showed comparable results to NGS, identifying 4 cases as high-risk for trisomy-21 and 26 cases as low-risk [28].

Chen et al. (2021) compared the results of segmental duplication quantification analysis using multiplex dPCR with routine NIPT employing NGS on 15 maternal plasma samples. Their findings indicated that dPCR yielded similar results to NGS, identifying 2 cases as trisomy 21 and 13 as euploids [29].

Dai et al. (2022) devised a dPCR protocol for cff-DNA enrichment in maternal plasma from normal pregnancies and evaluated its clinical utility in NIPT for trisomy 21 using 283 high-risk maternal plasma samples. According to their results, dPCR successfully detected all 25 cases of confirmed fetal trisomy 21; however, 7 FP results were also observed [25].

Laššáková et al. (2023) employed dPCR on plasma samples from 42 mothers at high risk for fetal aneuploidies to determine an optimal cut-off for NIPT. Utilizing this protocol, they successfully identified trisomy 21 in 30 high-risk maternal samples, accurately detecting the 6 trisomy 18 cases with dPCR and reporting no FPs [26].

Table 2 provides data regarding the performance of dPCR in non-invasive prenatal screening of trisomy-21.

**Table 2:** Summary of findings of the included studies on the utility of dPCR in trisomy-21 screening^a^

Study	Included cases		Digital PCR results			
	**Euploid 21**	**Trisomy 21**	**TN**	**TP**	**FN**	**FP**
Lee (2015) [1]	33	10	33	9	1	0
El Khattabi (2016) [2]	192	21	192	21	0	0
Xu (2016) [3]	12	3	12	3	0	0
Lee (2018) [4]	827	50	824	50	0	3
Wu (2018) [5]	78	28	78	25	3	0
Tan (2019) [6]	26	4	26	4	0	0
Chen (2021) [7]	13	2	13	2	0	0
Dai (2022) [8]	257	25	250	25	0	7
Laššáková (2023) [9]	24	6	24	6	0	0

*Abbreviations*: *FN* False negative, *FP* False positive, *NA* Not available, *TN* True negative, *TP* True positive

^a^Categorial data are presented as numbers

#### Trisomy 13 and 18

Dai et al. (2022) developed a dPCR protocol for cf-DNA enrichment in maternal plasma from normal pregnancies. They assessed multiplex dPCR's clinical utility in NIPT for trisomy 13 and 18 using 283 high-risk maternal plasma samples. The dPCR results were compared to confirmed fetal diagnoses via karyotyping, demonstrating 100.0% sensitivity and 98.2% specificity for trisomy 13 (TP = 1, TN = 276, FN = 5, FP = 0) and 90.0% sensitivity and 99.6% specificity for trisomy 18 (TP = 9, TN = 271, FN = 1, FP = 1) [25].

Laššáková et al. (2023) applied dPCR to plasma samples from 42 mothers (26 euploid) at high risk for fetal aneuploidies to establish an optimal cut-off for NIPT. Using this protocol, they successfully detected trisomy 18 in 30 high-risk maternal samples, accurately identifying the lone trisomy 18 case (confirmed via karyotyping analysis) with dPCR and reporting no FPs [26].

### Quality assessment

#### Risk of bias

Two studies were at high risk of bias [21, 25] and 4 were at unclear risk of bias [22, 24, 26, 28] for patient selection, as they did not enroll a consecutive or random sample of patients. The remaining 3 studies were considered at low risk of bias in patient selection [23, 27, 29]. One study was characterized by an unclear risk of bias in the index test domain due to the absence of information regarding the blinding of assessors to the reference test results [22]. In contrast, the remaining 8 studies demonstrated a low risk of bias concerning the index test [21, 23–29].

Three studies were rated with high bias for using NGS instead of karyotyping as the reference standard for aneuploidy detection [21, 28, 29]. The other 6 studies were judged to be at low risk of bias for reference standards. Furthermore, all reviewed studies were at low risk for the flow and timing domain of QUADAS-2.

**Fig. 2:**
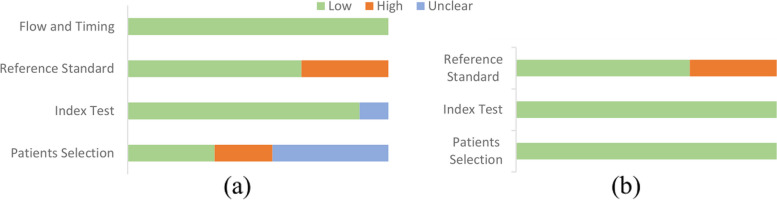
Proportion of studies with high, low, and unclear (**a**) risk of bias and (**b**) concerns regarding the applicability based on the QUADAS-2 statement

Collectively, 2 studies were considered at an overall low risk of bias, while the other 7 studies were at risk of bias. Table 3 and Fig. 2 (a) provide detailed information from the quality assessment of the included articles.

**Table 3:** Quality assessment of the included studies based on the QUADAS-2 statement

**Study**	**Risk of bias**				**Applicability concerns**		
	**Patient selection**	**Index test**	**Reference standard**	**Flow and timing**	**Patient selection**	**Index test**	**Reference standard**
Lee (2015) [1]							
El Khattabi (2016) [2]							
Xu (2016) [3]							
Lee (2018) [4]							
Wu (2019) [5]							
Tan (2019) [6]							
Chen (2021) [7]							
Dai (2022) [8]							
Laššáková (2023) [9]							1

High  Unclear  Low

#### Applicability

Except for 3 studies that were classified as having concerns regarding the applicability due to their utilized reference standards, the other 6 studies had low concerns regarding the applicability (see Table 3 and Fig. 2(b)).

### Meta-analysis

#### Trisomy 21

**Fig. 3:**
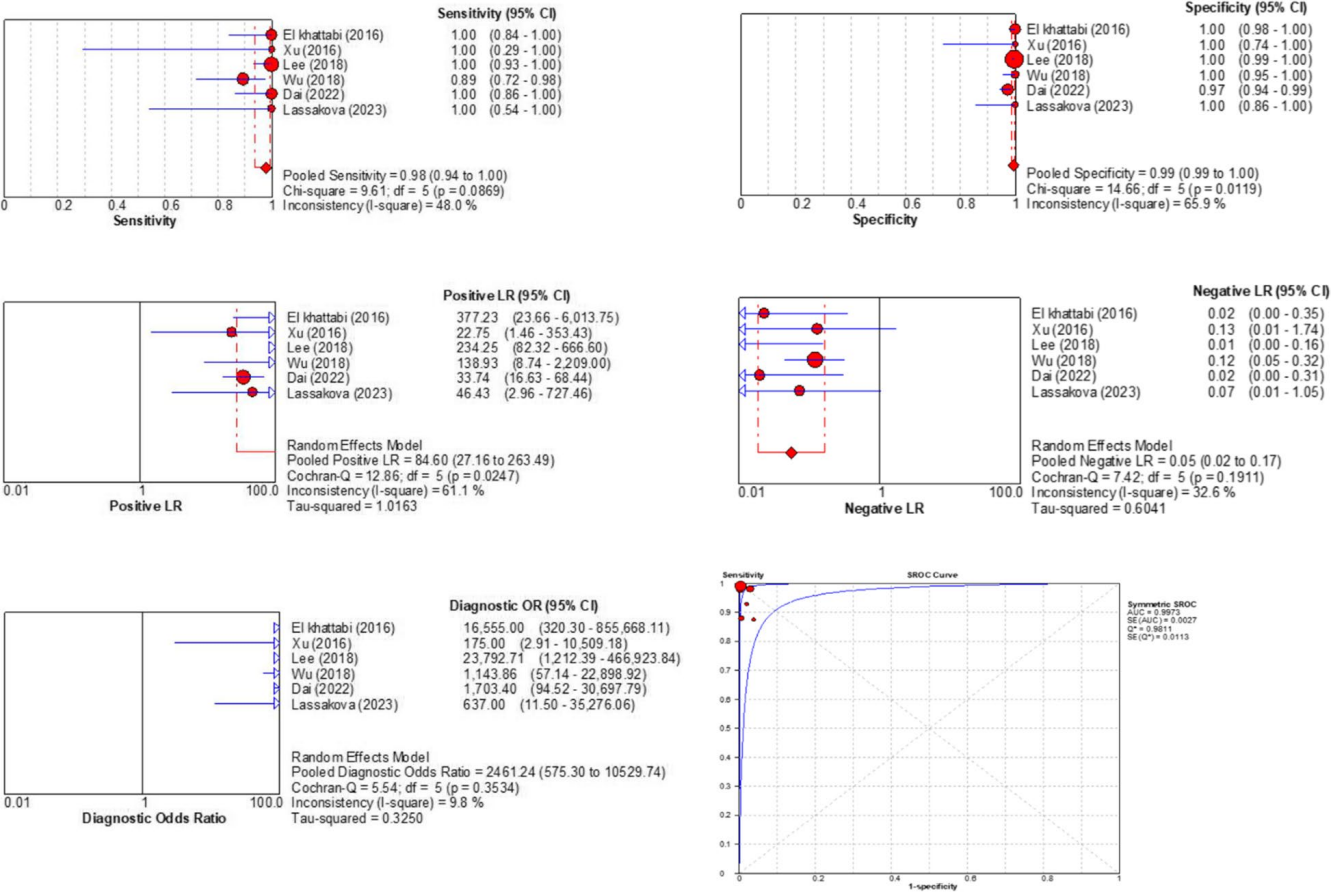
Forest plots for the pooled sensitivity, specificity, positive likelihood ratio, negative likelihood ratio, diagnostic odds ratio, and summary receiver operating characteristics for non-invasive prenatal screening of trisomy-21 using the dPCR

Nine studies assessed the utility of dPCR for non-invasive prenatal trisomy 21 screening; however, 3 studies compared the results of dPCR with NGS and were not included in our meta-analysis [21, 28, 29]. Consequently, the data from 6 studies that compared dPCR results with the karyotyping analysis results were pooled [22–27]. The pooled sensitivity and specificity were 98% [95%CI: 94–100] and 99% [95%CI: 99–100], respectively. The *I*^2^ and chi-square sensitivity values were 48% and 9.61, respectively, demonstrating a low heterogeneity between studies. However, there was a high heterogeneity in specificity values across the studies (*I*^2^ = 65.9% and chi-square = 14.66). The pooled PLR and NLR were 84.60 [95%CI: 27.16–263.49] and 0.05 [95%CI: 0.02–0.17], respectively, demonstrating that trisomy 21 fetuses were 84.60 times more likely than euploid fetuses to be detected by dPCR and there was a 0.05 chance of euploid cases being mistakenly screened as positive. The pooled DOR and calculated AUC were 2461.24 [95%CI: 575.30–10529.74] and 0.997, respectively, indicating a high overall accuracy for dPCR in non-invasive trisomy-21 screening (Fig. 3).

The results of our leave-one-out sensitivity analysis are provided in Table 4. By omitting each study, the pooled sensitivity ranged from 97 to 100%, which falls within the initially calculated 95% CI range. Additionally, the pooled specificity consistently remained at 99% after omitting each study. Furthermore, the calculated values of PLR, NLR, and DOR ranged from 52.55 to 162.16, 0.03 to 0.08, and 1356.4 to 3498.0, respectively, all of which lay within the initially calculated 95% CI ranges. These findings suggest that the results of our analyses remained consistent after omitting each article from the analysis.

**Table 4:** Results of the leave-one-out sensitivity analysis

Omitted study	Sensitivity [95% CI]; %	Specificity [95% CI]; %	PLR [95% CI]	NLR [95% CI]	DOR [95% CI]	sROC
El Khattabi (2016) [1]	97 [92–99]	99 [98–99]	70.22 [21.58–228.42]	0.06 [0.02–0.20]	1868.7 [392.8–8889.6]	0.997
Xu (2016) [2]	98 [93–99]	99 [98–100]	101.44 [28.32–363.35]	0.04 [0.01–0.16]	3498.0 [813.6–15,039.2]	0.998
Lee (2018) [3]	96 [90–99]	99 [97–100]	52.55 [18.48–149.39]	0.08 [0.04–0.19]	1356.4 [288.24–6328.7]	0.995
Wu (2018) [4]	100 [96–100]	99 [98–100]	79.64 [22.53–281.53]	0.03 [0.01–0.12]	2900.6 [485.5–17,330.8]	0.996
Dai (2022) [5]	97 [92–99]	99 [99–100]	162.16 [70.48–373.09]	0.06 [0.02–0.21]	2614.5 [411.64–16,606.5]	0.998
Laššáková (2023) [6]	97 [93–99]	99 [98–100]	92.46 [25.16–339.79]	0.05 [0.01–0.18]	2939.3 [556.9–15,512.4]	0.997

*Abbreviations*: *CI* Confidence interval, *DOR* Diagnostic odds ratio, *NLR* Negative likelihood ratio, *PLR* Positive likelihood ratio, *sROC* Summary receiver operating characteristics

#### Trisomy 13 and 18

Conducting a meta-analysis on dPCR accuracy in trisomy 13 and 18 screening was impractical due to the limited number of available studies.

## Discussion

Our conducted meta-analysis revealed notably high sensitivity and specificity for dPCR in the context of non-invasive prenatal screening for trisomy 21. While a meta-analysis was not feasible for trisomy 13 and trisomy 18 due to the limited number of available studies, the reported values of sensitivity and specificity for dPCR in screening for both aneuploidies were favorable. These outcomes suggest that dPCR can be regarded as a reliable tool for NIPT targeting autosomal aneuploidies. Furthermore, considering its lower cost and relatively shorter processing time, dPCR may demonstrate the potential to replace NGS in the realm of NIPT [9].

Recently, NIPT methods have gained significant popularity for screening fetal aneuploidies due to their feasibility and reliable performance in detecting chromosomal abnormalities. NIPT significantly reduces false positive results, thereby decreasing the need for further invasive diagnostic procedures such as amniocentesis or CVS, which are associated with an increased risk of adverse events like miscarriage [30, 31]. Furthermore, while routine first- and second-trimester screening methods, encompassing serum biomarkers and ultrasound, can identify abnormalities associated with aneuploidies, the growing awareness of their comparatively reduced sensitivity and accuracy has prompted an augmented scholarly focus on non-invasive genetic testing utilizing cff-DNA [32]. This burgeoning trend underscores the increasing acknowledgment of cf-DNA screening as a safer and more precise alternative within the domain of prenatal screening [33–35].

The predominant method for screening cf-DNA is NGS, renowned for its exceptional ability in detecting aneuploidies [26]. Recent studies have showcased a sensitivity range of 99.3–99.4%, 97.4–97.7%, and 90.6–97.5% for NGS in detecting trisomy 21, 18, and 13, respectively [36–39]. These findings underscore the reliability of NGS in clinical applications [40, 41]. However, the widespread adoption of NGS as a routine laboratory screening test faces challenges due to its time-consuming, complex, and expensive nature, thereby limiting its implementation [7, 42].

To address these challenges, dPCR emerges as a viable alternative to NGS [26]. In comparison to NGS, dPCR exhibits comparable sensitivity, and also offers a more straightforward procedure, accessible data analysis, and demands less labor and time (typically 2–3 h), making it a more cost-effective option [7]. Moreover, 3 articles reviewed in our study reported relatively similar results for dPCR and NGS in the non-invasive screening of trisomy 21 [21, 28, 29]. Collectively, these findings suggest that dPCR could serve as a practical substitute for NGS in NIPT for autosomal aneuploidies.

The expedited results and less complex technology associated with dPCR may contribute to its potential popularity, particularly in low- to middle-income countries with limited resources where NGS may not be readily available [43]. The significance of the rapid results provided by dPCR is particularly important in regions where early termination of pregnancy is subject to locally specified threshold regulations. Compared to first-trimester screening and NIPT, dPCR offers quicker results, enabling earlier termination and thereby reducing complications as well as social and psychological burdens for patients. However, it is important to note that while NGS can detect a broader range of causative sequences and has established clinical utility [44], further research is warranted to ascertain the comparative advantages of dPCR over NGS in NIPT.

The findings of our study should be interpreted with caution, considering its inherent limitations. Firstly, a considerable proportion of the studies included in our analysis featured a relatively limited number of maternal samples, thereby potentially compromising the generalizability of their results. Secondly, the maternal samples were sourced from a heterogeneous population of mothers across the studies. Notably, while some studies focused on mothers identified as high risk for aneuploidies in their routine prenatal evaluations, others included a broader spectrum of mothers with varying risks for fetal aneuploidies. This heterogeneity can significantly impact the synthesis and interpretation of findings from the included studies. Third, the methodological approaches employed for dPCR varied among the included studies, with some utilizing a single-plex approach and others employing a multiplex approach. Additionally, the target genes chosen for analysis differed across studies. These variations emphasize the necessity for future investigations to delineate the distinctions between different dPCR methods and elucidate their respective advantages and disadvantages in NIPT for fetal aneuploidies. Consequently, there remains a critical need for future studies with larger sample sizes encompassing diverse groups of mothers and employing various methodological approaches to definitively determine the clinical utility of dPCR in the non-invasive prenatal screening of aneuploidies. Furthermore, our quality assessment indicated a high risk of bias in most of the reviewed articles. This highlights the need for future studies with more standardized methodologies to produce more reliable findings regarding the clinical utility of dPCR in NIPT. Lastly, it is crucial to note that only two studies provided data on the performance of dPCR in non-invasive prenatal screening of trisomy 13 and 18, rendering it impractical for us to conduct a meta-analysis. Despite the promising results reported in these studies, further research in these specific areas is imperative.

## Conclusions

Our study revealed a favorable performance for dPCR in the non-invasive prenatal screening of trisomy 21. Although the available data on the utility of dPCR for trisomy 13 and 18 were limited, the reported accuracies were promising. These collective findings suggest that dPCR holds promise as a potential alternative to NGS for autosomal aneuploidies, given its favorable efficiency, rapid procedural timeline, and lower cost. Nevertheless, further research in this field is imperative to demonstrate the clinical utility of dPCR in non-invasive aneuploidy screening and to elucidate its advantages over NGS.

### Supplementary Information

Supplementary Material 1. Searched keywords and utilized filters within each dataset.

Supplementary Material 2. PRISMA 2020 checklist.

## Data Availability

This review article does not include specific data for sharing, as its focus is on summarizing and analyzing existing literature rather than presenting original research findings.

## Abbreviations

AUC: Area Under the Curve
Cff-DNA: Cell-free fetal deoxyribonucleic acid
CI: Confidence Interval
CVS: Chorionic Villous Sampling
DNA: Deoxyribonucleic acid
DOR: Diagnostic Odds Ratio
dPCR: Digital polymerase chain reaction
FN: False negative
FP: False positive
NGS: Next-Generation Sequencing
*I*^2^: Inconsistency Index
NIPT: Non-Invasive Prenatal Testing
NLR: Negative Likelihood Ratio
PCR: Polymerase chain reaction
PLR: Positive Likelihood Ratio
PRISMA: Systematic Reviews and Meta-Analysis
qPCR: Quantitative PCR
QUADAS-2: Quality Assessment of Diagnostic Accuracy Studies-2
sROC: Summary Receiver Operating Characteristics
TN: True Negative
TP: True Positive
