# Supplementary material for: Potential efficacy of digital polymerase chain reaction for non-invasive prenatal screening of autosomal aneuploidies: a systematic review and meta-analysis
Source: BMC Pregnancy Childbirth. 2024 Jul 11;24:472. doi: 10.1186/s12884-024-06655-0 (PMC11238349; doi:10.1186/s12884-024-06655-0)
Supplement: Supplementary file 1 — Supplementary Material 1. Searched keywords and utilized filters within each dataset. [file 12884_2024_6655_MOESM1_ESM.docx]

1. PubMed (79 records):

| Search Keywords | filters |
| --- | --- |
| ("Digital Polymerase Chain Reaction" OR "Digital PCR" OR "DigitalPCR" OR "Digital Droplet Polymerase Chain Reaction" OR "Digital Droplet PCR" OR " Droplet Digital Polymerase Chain Reaction" OR " Droplet Digital PCR" OR "ddPCR" OR "dPCR" OR "dePCR") AND ("Aneuploidy"[Mesh] OR "Aneuploidy" OR "Aneuploidies" OR "Aneuploid" OR "Trisomy"[Mesh] OR "Trisomy" OR "Trisomy 13 Syndrome"[Mesh] OR "Trisomy 13" OR "Patau Syndrome" OR "Patau's Syndrome" OR "Pataus Syndrome" OR "Trisomy 13 Syndromes" OR "Trisomy 18 Syndrome"[Mesh] OR "Trisomy 18" OR "Trisomy E Syndrome" OR "Edwards Syndrome" OR "Down Syndrome"[Mesh] OR" Down Syndrome" OR "Down's Syndrome" OR "Downs Syndrome" OR "Mongolism" OR "Trisomy 21") | Search keywords were conducted through “All Fields”, with no filters. |

2. Web of Science (153 records):

| Search Keywords | filters |
| --- | --- |
| ("Digital Polymerase Chain Reaction" OR "Digital PCR" OR "DigitalPCR" OR "Digital Droplet Polymerase Chain Reaction" OR "Digital Droplet PCR" OR " Droplet Digital Polymerase Chain Reaction" OR " Droplet Digital PCR" OR "ddPCR" OR "dPCR" OR "dePCR") AND ("Aneuploidy"[Mesh] OR "Aneuploidy" OR "Aneuploidies" OR "Aneuploid" OR "Trisomy"[Mesh] OR "Trisomy" OR "Trisomy 13 Syndrome"[Mesh] OR "Trisomy 13" OR "Patau Syndrome" OR "Patau's Syndrome" OR "Pataus Syndrome" OR "Trisomy 13 Syndromes" OR "Trisomy 18 Syndrome"[Mesh] OR "Trisomy 18" OR "Trisomy E Syndrome" OR "Edwards Syndrome" OR "Down Syndrome"[Mesh] OR" Down Syndrome" OR "Down's Syndrome" OR "Downs Syndrome" OR "Mongolism" OR "Trisomy 21") | Search keywords were conducted through “All Fields”, with no filters. |

3. Embase (192 records):

| Search Keywords | filters |
| --- | --- |
| ("Digital Polymerase Chain Reaction" OR "Digital PCR" OR "DigitalPCR" OR "Digital Droplet Polymerase Chain Reaction" OR "Digital Droplet PCR" OR " Droplet Digital Polymerase Chain Reaction" OR " Droplet Digital PCR" OR "ddPCR" OR "dPCR" OR "dePCR") AND ("Aneuploidy" OR "Aneuploidies" OR "Aneuploid" OR "Trisomy" OR "Trisomy 13" OR "Patau Syndrome" OR "Pataus Syndrome" OR "Trisomy 13 Syndromes" OR "Trisomy 18" OR "Trisomy E Syndrome" OR "Edwards Syndrome" OR " Down Syndrome" OR "Downs Syndrome" OR "Mongolism" OR "Trisomy 21") | Search keywords were conducted through “Broad Search”, with no filters. |
